# The Work Ability of Hong Kong Construction Workers in Relation to Individual and Work-Related Factors

**DOI:** 10.3390/ijerph15050990

**Published:** 2018-05-14

**Authors:** Jacky Y. K. Ng, Alan H. S. Chan

**Affiliations:** Department of Systems Engineering and Engineering Management, City University of Hong Kong, Hong Kong, China; alan.chan@cityu.edu.hk

**Keywords:** work ability, work ability index, construction workers, ageing

## Abstract

The shortage in Hong Kong of construction workers is expected to worsen in future due to the aging population and increasing construction activity. Construction work is dangerous and to help reduce the premature loss of construction workers due to work-related disabilities, this study measured the work ability of 420 Hong Kong construction workers with a Work Ability Index (WAI) which can be used to predict present and future work performance. Given the importance of WAI, in this study the effects of individual and work-related factors on WAI were examined to develop and validate a WAI model to predict how individual and work-related factors affect work ability. The findings will be useful for formulating a pragmatic intervention program to improve the work ability of construction workers and keep them in the work force.

## 1. Introduction

### 1.1. Work Ability of Construction Workers and Other Occupations

Because construction workers are required to perform physically demanding and repetitive work, they are exposed to higher risk of work-related disabilities than workers in other industries [1,2]. With ageing, there will be a general deterioration in physical capacity and an increase in the risk of work-related disabilities [3]. Occupational disability has been found to be the prime reason contributing to the early dropout of construction workers worldwide [4]. To reduce the premature loss of construction workers as a result of work-related disability, the concept of work ability measurement has been increasingly used in recent years as an important tool to develop suitable interventions [5,6,7,8].

### 1.2. Work Ability

The term “work ability” was first introduced in Finland in the 1980s and was defined as: “how good is the worker at present and in the near future, and how able is he/she to do his/her job with respect to work demands, health, and mental resources?” [9]. Thus, work ability is the result of the interaction between the worker and his/her work, as well as a measure of the balance between a worker’s resources and work demands, both physical and mental. The maintenance and promotion of suitable work ability has been consistently reported to have a significant and positive socio-economic impact on society in terms of increased productivity and decreased early retirements [10]. In contrast, poor work ability has been associated with increased absences because of sickness, reduced productivity, and increased early retirements [11]. Therefore, it is of critical importance to identify the factors that affect work ability.

### 1.3. Work Ability Index

In order to quantify work ability and provide a work capacity indicator for existing workers, the Work Ability Index (WAI) was developed by Finnish researchers [12,13]. Since then, WAI has been widely adopted as a useful means of work ability assessment for workplace health prevention. WAI consists of seven distinct dimensions and is derived as the sum of these dimensions. The range of WAI scores is from 7 to 49, and work ability scores are classified as: excellent (44–49), good (37–43), moderate (28–36) and poor (below 28) [12,14,15]. Ilmarinen and Tuomi [9] found that workers with high WAI scores generally have a low incidence of premature retirement or dropout, and a better quality of life.

### 1.4. Individual and Work-Related Factors Affecting Work Ability

Past studies have reported that work ability can be affected by individual and work-related factors [16,17]. Regarding individual factors, demographic characteristics, lifestyle, health-related factors and individual competencies are possible factors that may lead to variations in work ability. For demographic characteristics, age was found to be a significant factor contributing to decline in work ability [13,18,19,20] and the decline was more significant in workers in physically demanding jobs than for those in mentally demanding jobs [13]. In terms of gender, females were found to have significantly lower work ability than males among nursing personnel [21]. Body mass is another significant factor for work ability, and Laitinen et al. [22] found that normal weight adults were more likely to have better work ability than thin and obese individuals. Bridger and Bennett [23] found that body mass index (BMI) was negatively associated with the work ability of seafarers, which is generally a moderately demanding occupation.

As for the effect of lifestyle on work ability, having a smoking habit was associated with a lower WAI among municipal employees in Finland and Poland [24]. However, such an association has been reported to be inconsistent in several cross-sectional studies [25,26]. Similarly, there has been no consensus on the effect of alcohol consumption on the WAI for Dutch construction workers [5,27,28]. Insomnia and objective short duration of sleep were reported to be both independently associated with poor work ability in a recent study by Lian et al. [29]. Frequency of leisure time physical activity was another significant factor for work ability, and it was found that participants who reported higher levels of leisure time physical activity had a higher chance of having better work ability [30].

For the health-related factors, self-perceived poor health was found to be the strongest factor associated with poor work ability, and was significant even after controlling for age. This association with lower work ability has been shown to extend to workers with musculoskeletal and psychosomatic symptoms [19,31]. Also, the presence of musculoskeletal disorders (MSDs) negatively affected several aspects of work ability [32].

Individual (or professional) competence is one of the dimensions featured in the work ability house model of Ilmarinen [33]; however, there has been little if any study of its impact on WAI scores. Continuous development of professional knowledge and competence is a very important prerequisite for work ability in order to meet changes in challenges and demands at work. Given that there is limited knowledge of the impact of professional competence on work ability, it has been included in the proposed WAI conceptual model to be used in this study, to understand its contribution to variations in WAI scores.

Apart from individual factors, work-related factors have been reported to significantly affect WAI. A recent review conducted by van den Berg et al. [34] revealed that the majority of relevant studies found that lower WAI scores were associated with high physical and psychological demands. A mismatch between work demands and individual physical capacity has been found to increase the chance of acquiring MSDs [35], subsequently reducing work ability and possibly leading to long-term sickness absence and early retirement [5,36].

According to the Job Demand-Control (-Support) model [37], three important factors, namely, work demand, job control, and social support, determine workplace qualities. In the model, with reference to strain hypothesis, workers exposed to high work demand and low-control jobs are likely to suffer from high strain level, whereas those exposed to low-demand and high-control jobs generally have low strain level [38]. Social support was later added to the model to expand the strain hypothesis to iso-strain hypothesis. Findings showed that high-demand, low-control, and low-support jobs lead to high levels of anxiety and depression [39]. However, the relationships of these three workplace factors with WAI have never been assessed in past studies. It is of great interest to examine how workplace qualities affect work ability.

### 1.5. Past Studies on WAI

Table 1 summarizes several WAI studies measuring work ability for different occupations in different countries. The average WAI for different workers in different studies were generally regarded as “good” (37–43). However, no consensus has been reached on the association between the type of work and the level of work ability. For instance, white-collar workers do not always have better work ability than blue-collar workers. For example, in the study by Alavinia et al. [5], Dutch construction workers generally had higher WAI than Chinese professionals and clerical workers in the study of Lin et al. [40], implying that the work ability of a worker can be influenced by many different factors. Work-related factors and individual lifestyle factors both contribute to the differences found in WAIs.

### 1.6. Aims of This Study

Given that Hong Kong is very likely to suffer from an unprecedented manpower shortage in the coming decades, and the number of older workers having to consider extending their working years will increase for various reasons, the major one being financial problems that they would normally extend their working life to earn more saving to support their living after retirement. Therefore, the primary aims of this study were to examine the work ability of Hong Kong construction workers and to develop and validate a WAI conceptual model for predicting how individual and work-related factors affect the work ability of construction workers. Path analysis was used to test the WAI model and to provide explanations for the casual relations (direct and/or indirect) among the variables in the model and their relative importance. Goodness-of-fit statistic was calculated to examine the fit of the model, and the model was used to predict regression weight. The more information available, the more confident we can be that proposed work ability interventions will be successful, and that promotion programs among construction workers in Hong Kong will sustain this valuable workforce and prevent premature dropouts.

### 1.7. WAI Conceptual Model and Hypotheses

**Figure 1:**
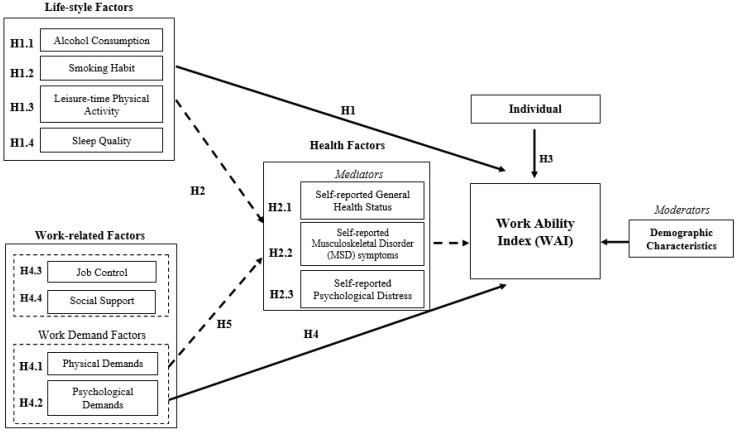
The conceptual work ability model in the present study (Solid lines are direct effects and dashed lines are indirect effects).

Figure 1 shows the WAI conceptual model that was developed and investigated in this study. For achieving the stated aims, the following hypotheses were formulated in order to study the direct and indirect effects of the selected individual and work-related factors on the work ability of Hong Kong construction workers.

**Hypothesis 1** **(H1).** Lifestyle factors are associated with the WAI.

H1.1. Alcohol consumption is negatively associated with the WAI.

H1.2. Smoking habit is negatively associated with the WAI.

H1.3. Leisure-time physical activity is positively associated with the WAI.

H1.4. Sleep quality is positively associated with the WAI.

**Hypothesis 2** **(H2).** The associations between the lifestyle factors and the WAI are mediated by health-related factors.

H2.1. Self-reported general health status is positively associated with the WAI.

H2.2. Self-reported MSD symptoms are negatively associated with the WAI.

H2.3. Self-reported psychological distress is negatively associated with the WAI.

**Hypothesis 3** **(H3).** Individual competence is positively associated with the WAI.

**Hypothesis 4** **(H4).** Work demand factors are associated with the WAI.

H4.1. Physical demands are negatively associated with the WAI.

H4.2. Psychological demands are negatively associated with the WAI.

H4.3. Job control is positively associated with the WAI.

H4.4. Social support is positively associated with the WAI.

**Hypothesis 5** **(H5).** The associations between the work-related factors and the WAI are mediated by individual health-related factors.

In fact, work demands, social support and job control examined in this study are linked with psychosocial hazards at the workplace. Psychosocial hazards can be regarded as the aspects of the work design and management as well as the social and organizational context of the work that potentially leading to psychological or physical harm to workers [52]. Therefore, those factors should be included in the study and formulation of intervention programs for improvement in work ability of construction workers by minimizing their exposure to psychosocial risks at work.

## 2. Methods

### 2.1. Participants

A total of 420 Hong Kong construction workers were interviewed. Among them, 385 (91.67%) were male and 35 (8.33%) were female, with ages ranging from 31 to 50 years old. Among the female participants, most of them were laborer or cleaner (13) followed by building service technician (9). The pattern of working hours of the construction industry is quite stable, such that workers usually work from 8 a.m. to 5 p.m. for 5 to 6 days per week. The construction workers were reached with the assistance of 20 trade unions and 60 construction companies and had diverse demographic backgrounds, which made the sample representative to the construction workforce in Hong Kong.

### 2.2. Procedure

All participants gave informed consent before the start of the interview. They were informed about the purpose of the study and the use of the data, and they could withdraw from the interview at any time without providing any reason. Interviews were conducted by three different interviewers individually when the construction workers were paying visits to the amenity or resting facilities of the supporting organizations. The whole data collection process was not influenced or interfered by the supporting organizations. All the respondents voluntarily participated in the survey with no presence of staff of the supporting organizations, or people other than the research staff. They were assured that the whole study was conducted by university staff with a high level of data anonymity, security and confidentiality. The interviewer adopted a face-to-face approach to read aloud the questions one by one according to the sequence listed in the questionnaire. The participants’ responses were then marked on the questionnaire by the interviewer. The interviews were conducted in Cantonese and lasted for approximately 15 min for each participant.

### 2.3. Materials

The interview survey measurement consisted of three major sections: (1) WAI measurement; (2) individual factors; and (3) work-related factors. The questionnaires were translated from English into Chinese by a back-translation method. With this method, the English questionnaire was first translated into Chinese by a translator and then translated back into English by another translator who was blinded to the original questionnaire. The original and the translated questionnaires were then compared. For any discrepancy, a safety and health professional was asked to determine which wording was better to reflect the content, given the fact that the work ability model itself is relying on occupational health and safety. In addition, a pilot study was also conducted with a few construction workers to ensure that they understood the meaning of the questions.

#### 2.3.1. Work Ability Index (WAI)

Work ability was measured by the Work Ability Index (WAI) questionnaire developed by Ilmarinen and his colleagues [13,15].

#### 2.3.2. Individual Factors

The individual factors examined in this study were: lifestyle factors, health-related factors, individual competence and the demographic factors for the workers.

##### Lifestyle Factors

There were four questions concerning the lifestyle of the workers. The questions covered smoking habit and alcohol consumption, sufficiency of sleep and frequency of leisure-time physical activity. The questions regarding alcohol consumption were: What is the average amount of alcoholic drinks you consume per week? How many years have you been drinking alcohol? The questions regarding smoking were: How many cigarettes do you smoke per day? How many years have you been smoking? The question “Did you have enough sleep in the past month?” was used to measure the sleep quality. For leisure-time physical activity, two questions “How much did you move about physically in your spare time?” and “How hard did you exert yourself physically in your spare time?” were asked and participants responded to these two questions using a 5-point Likert scale. The scale for Frequency of physical activity was 1: seldom, 5: always, and the scale for strength of physical activity was 1: extremely low, 5: extremely high. 

##### Health-Related Factors

For the health-related factors, measuring physical capacity (e.g., cardiorespiratory capacity and musculoskeletal capacity) and psychological capacity (e.g., perceptual and conceptual abilities) of construction workers through the various tests and measurements required was impractical. Therefore, self-reports on their general health status, musculoskeletal disorders (MSDs), and psychological distress were used for individual physical and psychological capacity evaluation. The general health status of the workers was assessed by requiring them to answer two questions, one was on how they perceive their own health conditions and, the other was about their health conditions compared with those of others in the same age group. Both questions were measured using a five-point Likert-type scale (1: very poor, 5: very good). MSD symptoms were measured using a modified Nordic questionnaire that contained three questions regarding nine anatomic positions (i.e., neck, shoulder, upper back, lower back, elbow, wrist/hand, hip/thigh, knee, feet) of the workers [53,54]. For instance, the participants were asked “During the last 12 months have you had a job-related ache, pain, discomfort?”, “During the last 12 months have you been prevented from doing your day’s work due to this condition?”, and “During the last 12 months have you seen a physician for this condition?” for each of the nine anatomic positions. For the self-reporting of psychological distress, the Kessler Psychological Distress Scale (K6) [55] was used. The scale contains six questions about the emotional state of the worker. For example, “In the past 4 weeks, about how often did you feel nervous?”. Each question is rated on a five-point Likert-type scale where 5 represented “most of the time” and 1 represented “no time”.

##### Individual Competence

For the measurement of individual competence, the Career Competencies Questionnaire (CCQ) developed and validated by Akkermans et al. [56] was adopted. The questionnaire contained 21 items measuring six factors concerning career competence, namely, reflection on motivation, reflection on qualities, networking, self-profiling, work exploration, and career control. For example, one of the questions “I know my strengths in my work.” was asked for the factor of “Reflection on qualities”. CCQ was chosen because it has considered different aspects of professional competence, which can provide a solid reference for assessing the skills and knowledge, motivation, social network, career control of the participants towards their job. Also, CCQ has been well validated and applied to assess the professional competence of workers in different fields. Hence, it is a reliable measurement tool for examination of the relationship between work ability and professional competence. All of the items were measured on a five-point scale with 1 representing “completely disagree” and 5 representing “completely agree”.

##### Demographic Characteristics

The demographic factors of age, gender, height, weight, marital status, education level, job category, family scenario, and anticipated age of retirement were included in the questionnaire. BMI was calculated to determine whether the participants were normal (<25 kg/m^2^), overweight (≥25 kg/m^2^), or obese (≥30 kg/m^2^) [57].

#### 2.3.3. Work-Related Factors

In this study, the Job Content Questionnaire (JCQ), which is a job demand measure that has been extensively used for many occupations, was used to assess the physical and psychological workloads of the construction workers. Ten items concerning perceived physical (5 items) and psychological (5 items) demands at work from the JCQ were used [58,59]. For example, questions of “I am often required to move or lift very heavy loads on my job.” and “I have enough time to get the job done.” were asked for evaluating the physical and psychological demands, respectively. The items were measured on a five-point Likert-type scale with 1 representing “strongly disagree” and 5 representing “strongly agree”. To measure the levels of job control and social support and their interactions with work demand, the Demand Control Support Questionnaire (DSCQ) [40] and JCQ [60] were used to assess workers’ perceived level of job control and social support at work, respectively. The DSCQ contained 5 items (e.g., Do you have the possibility to decide for yourself how to carry out your work?), and each item was scored on a four-point scale (1: totally disagree to 4: totally agree). Eight items were used from JCQ, four to measure supervisor support (e.g., My supervisor/company pays attention to what I am saying.) and four for co-worker support (e.g., When needed, my colleagues will help me.). Each item was scored on a five-point Likert-type scale (1: strongly disagree to 5: strongly agree).

#### 2.3.4. Internal Consistence of Different Measurements

Cronbach’s alpha values for the measurements of WAI, psychological distress, career competence, physical demands, psychological demands, decision latitude, supervisor/company support, and co-worker support were 0.62, 0.83, 0.94, 0.60, 0.66, 0.76, 0.89 and 0.90, respectively. Because all Cronbach’s alpha values were equal to or larger than 0.60, the internal consistency reliability for the different measurements was considered acceptable [61,62].

## 3. Results

Work ability index (WAI) scores for the 420 participants were obtained. The causal relations between WAI score and the individual and work-related factors were examined by the WAI conceptual model using Path Analysis. Cronbach’s alpha test was conducted to check the internal consistency of the items in each factor to ensure that they were measuring the same underlying construct.

### 3.1. Work Ability Index (WAI)

The average score for each subscale and the overall WAI score are shown in Table 2. The overall WAI score for this group of workers ranged from 27 to 49 with an average of 45.12. According to the WAI classification [excellent (44–49), good (37–43), moderate (28–36) and poor (below 28)], 72.62% of the participants here had excellent work ability, followed by good (25.71%), moderate (1.43%) and poor work ability (0.24%). The WAI for male participants was 45.14, which was slightly higher than that of female participants (0.45%). The difference was insignificant in the independent samples *t*-test (*p* > 0.05), implying that male and female construction workers had no significant difference in their self-reported work ability.

### 3.2. Lifestyle Factors

Table 3 shows the descriptive data for lifestyle factors, health factors, individual competence, and work-related factors.

#### 3.2.1. Alcohol Consumption

The participants consumed an average of 377.99 mL of alcohol per week, and they have been drinking alcohol for an average of 2.78 years (Table 3). Excluding the non-alcoholic drinkers, the alcohol consumed by the participants (*N* = 93) on average was 1707.04 mL per week and they had been drinking for an average of 12.56 years. Pearson’s correlations were calculated to evaluate the correlations between WAI and alcohol consumption. The results showed significant negative correlations between the WAI and the amount of alcohol consumption (r = −0.14, *n* = 420, *p* < 0.01), as well as between the WAI and the years of alcohol drinking (r = −0.12, *n* = 420, *p* < 0.05) (Table 4), indicating that the more alcohol the participants consumed per week and the longer the time they had been drinking alcohol, the lower their WAIs.

#### 3.2.2. Smoking Habit

The average number of cigarettes smoked by the participants was 3.77 per day, and the average number of years of smoking was 4.15 (Table 3). Excluding the non-smokers, the participants (*N* = 127) smoked an average of 12.46 cigarettes per day, and had been smoking for an average of 13.60 years. Correlation analysis showed significant negative correlations between the WAI and smoking habit. The more cigarettes that the participant smoked, the lower their WAI (r = −0.23, *n* = 420, *p* < 0.001), and the longer the years the participants smoked, the lower their WAI (r = −0.25, *n* = 420, *p* < 0.001) (Table 4).

#### 3.2.3. Leisure-Time Physical Activity

On a five-point Likert-type scale, the frequency of physical activity during leisure time was 3.29 (1: seldom, 5: always), and the strength of the physical activity was 3.15 (1: extremely low, 5: extremely high) (Table 3). Correlation analysis showed significant positive correlations between the WAI and both the frequency of physical activity (r = 0.26, *n* = 420, *p* < 0.001) and the strength of physical activity (r = 0.23, *n* = 420, *p* < 0.001) (Table 4).

### 3.3. Health-Related Factors

#### 3.3.1. General Health Status

The average value of self-reported general health status was 4.39 for the participants (Table 3). A significant positive correlation was found between the WAI and general health status (r = 0.59, *n* = 420, *p* < 0.001) (Table 4), indicating that the better the self-perceived general health status, the higher the WAI of the participants.

#### 3.3.2. MSD Symptoms

Self-reported MSD symptoms covered nine body positions of participants. The questions covered three levels of MSD symptoms that the participants suffered from in the previous 12 months, viz., mild (having job-related ache, pain, discomfort), moderate (prevented from doing day’s work), and severe (consulted a physician). The average number of mild, moderate and severe MSD symptoms was 1.08, 0.52 and 0.44, respectively. Overall, the participants suffered from 2.04 MSD symptoms (Table 3). Correlation analyses showed significant negative correlations between the WAI and mild (r = −0.43, *n* = 420, *p* < 0.001), moderate (r = −0.23, *n* = 420, *p* < 0.001) and severe (r = −0.43, *n* = 420, *p* < 0.001) levels of MSD symptoms (Table 4). These results implied that participants with a higher level of MSD symptoms will have lower WAI.

#### 3.3.3. Psychological Distress

On average, the score for psychological distress was 28.46 (Table 3). The lower the value, the more frequently the participant had experienced psychological distress during the previous 30 days. Correlation analysis showed a positive correlation between the WAI and psychological distress (r = 0.38, *n* = 420, *p* < 0.001) (Table 4), implying that participants suffering less psychological distress had higher WAI.

### 3.4. Individual Competence

Individual competence was measured by six underlying factors. The average score for individual competencies was 4.29 (Table 3). Correlation analysis showed a significant positive correlation between WAI and individual competence (r = 0.40, *n* = 420, *p* < 0.001) (Table 4), indicating that participants with a higher level of career competence had better work ability.

### 3.5. Work-Related Factors

#### 3.5.1. Physical Demands

The scores for physical demands ranged from 5 to 20, and the average value for physical demands was 12.93 (Table 3). A high score represents a high level of perceived physical demands. Correlation analysis showed a significant negative correlation between the WAI and physical demands (r = −0.13, *n* = 420, *p* < 0.01) (Table 4), implying that work that was perceived to be more physically demanding was associated with lower work ability.

#### 3.5.2. Psychological demands

The scores for psychological demands ranged from 12 to 46, with an average value of 23.04 (Table 3). A high score represents a high level of perceived psychological demands. Correlation analysis showed a significant negative correlation between the WAI and psychological demands (r = −0.52, *n* = 420, *p* < 0.001) (Table 4), indicating work that was perceived to be more psychologically demanding was associated with lower work ability.

#### 3.5.3. Job Control

Job control was measured by the factor of decision latitude. The possible range for decision latitude was 24 to 96, and the value for the participants ranged from 26 to 94. The average value for decision latitude was 74.68 (Table 3), and a significant positive correlation was observed between the WAI and the decision latitude (r = 0.34, *n* = 420, *p* < 0.001) (Table 4). The results indicated that participants who reported more job control at work had significantly better work ability.

#### 3.5.4. Supervisor/Company Support

The average score for supervisor/company support was 14.63, with the minimum and maximum values being 5 and 16, respectively (Table 3). Correlation analysis showed a significant positive correlation between the WAI and supervisor/company support (r = 0.39, *n* = 420, *p* < 0.001) (Table 4), indicating that participants reporting higher levels of supervisor/company support will have significantly better work ability.

#### 3.5.5. Co-Worker Support

The range for co-worker support was 4 to 16, and the average value was 13.83 (Table 3). Correlation analysis showed a significant positive correlation between WAI and co-worker support (r = 0.33, *n* = 420, *p* < 0.001) (Table 4), indicating that participants reporting more co-worker support will have better work ability.

### 3.6. Path Analysis

**Figure 2:**
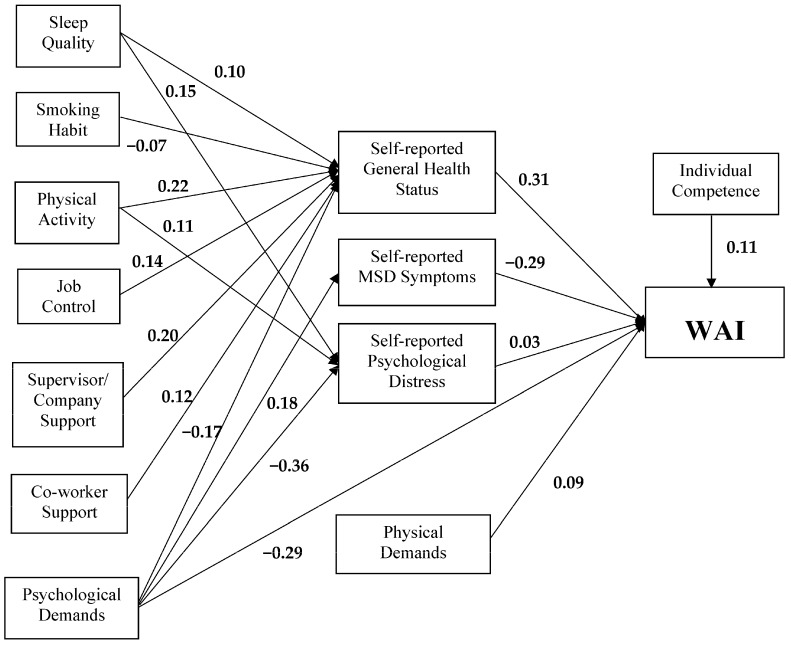
Path analysis for the WAI model (Only the significant paths are shown). Regression coefficients are shown on the respective paths.

Path analysis was used for mapping any direct and indirect relationships between the variables tested, to identify the strength of the relationships and the goodness of fit of the WAI model. The hypothesized model was fitted to the data and the model fit was good with the fit indexes meeting the statistical criterion. The Comparative Fit Index (CFI) (0.94) was larger than 0.90 and both Standardized Root Mean Square Residual (SRMR) (0.036) and Root Mean Square Error of Approximation (RMSEA) (0.066) were smaller than 0.10. Chi-square statistic was non-significant and the model explains 50% of the variance in WAI score (R^2^ = 50%) (Figure 2).

As shown in previous sections, sleep quality, smoking habit, leisure-time physical activity, job control, supervisor/company support, co-worker support, and psychological demands all directly influenced the WAI. However, in this study, these effects were mediated by general health status, MSD symptoms, and psychological distress, and Figure 2 shows the paths of these mediated effects on WAI through the health-related factors. For sleep quality, its effect on the WAI was completely mediated by general health status and psychological distress. Simply put, the variance of sleep quality is accounted for by the mediator general health status. A similar pattern was found for leisure-time physical activity, for which general health status fully mediated the effect of leisure-time physical activity on the WAI. For the rest of the variables, viz., smoking habit, job control, supervisor/company support, and co-worker support, it was found that general health status fully mediated their effects on the WAI. Path analysis also revealed that the effect of psychological demands on the WAI was partially mediated by general health status, MSD symptoms, and leisure-time physical activity separately. Despite the mediation of general health status, MSD symptoms, and leisure-time physical activity, it was found that psychological demands still directly influenced the WAI. Moreover, physical demands and individual competencies both had significant direct effects on the WAI.

Path analysis also revealed significant mediation effects on job control, supervisor/company support, and co-worker support on the WAI through general health status. The results of the hypothesis testing are summarized in Table 5. Other hypotheses were confirmed in this study, except for the lifestyle factor of alcohol consumption (H1.1).

## 4. Discussion

### 4.1. Comparison of Construction Worker WAIs in Different Places

The average WAI score in this study was relatively higher than those found in previous studies on construction workers in Western countries [5,48]. The higher average WAI of the participants in this study may be partly due to the fact that construction workers in Hong Kong are generally paid a daily rate. Therefore, they are less willing to request sick leave and may be more likely to present a positive picture of themselves particularly when responding to WAI items such as “work impairment due to diseases”. Such a tendency was reflected by the relatively high scores in the relevant WAI sub-questions: number of current diseases diagnosed by a physician, estimated work impairment due to diseases, and sick leave during the past year. Notably, the average body mass index (BMI) of the participants in this study was 22.9, whereas those in the studies of Alavinia et al. [5,29] were 26.5 and 26.2, respectively. Previous findings have found that high BMI was associated with low work ability [17,24]. The average BMI of the construction workers in this study was much lower than those found in several Western studies where many of the BMIs were classified as overweight or obese; hence, a higher average WAI score was obtained here.

### 4.2. Individual Factors

Several studies have shown that age is a significant factor leading to a decline in work ability [12,19,20]. However, in the current study, age did not significantly affect the WAI score after controlling for the effects of other demographic variables. This result may be due to the relative youth of the study population in the current work, with 79.76% of the participants being below the age of 51; and it has been shown that work ability decreased after the age of 51 [13,19,63]. However, in this study, there was no significant effect of age on the WAI score after controlling for the effect of number of working years, which implies that the association between age and the WAI might be confounded by number of years worked. A similar result was reported by Ghaddar et al. [64]. Work tasks in the construction industry are often repetitive and physically demanding, so much so that the greater the number of years working in the industry, the higher the chance of suffering from occupational disability [1,2]. Also, here, BMI was a significant factor affecting participants’ work ability. Participants with BMI of over 25 (i.e., obese) had significantly lower work ability than those with lower BMI. Similar findings have been reported by previous research [24].

### 4.3. Health-Related Factors

As outlined in the proposed conceptual work ability model, self-reported general health status, self-reported MSD symptoms, and self-reported psychological distress are regarded as the health-related factors having mediating effects between lifestyle factors and work ability, as well as between work-related factors and work ability. The findings of this study indicated that, for lifestyle factors, the effects of sleep quality, smoking habit, and leisure-time physical activity on WAI scores were fully mediated by self-reported general health status. Self-rated health status has been shown to be a valid health measure in previous studies [65,66], and it has been found to have a strong association with work ability, such that individuals who perceived their health as poor were more likely to have poorer work ability than others who perceived their health as moderate or good [19]. In the current study, participants reporting sufficient sleep, fewer years of smoking, or lower frequency of physical activity had better general health status and thereby better work ability.

### 4.4. Lifestyle Factors

The findings of this study show that the positive impact of sleep quality on work ability was fully mediated by self-reported general health status. Sleepiness and sleep quality have been reported to be crucial factors affecting general health status [67]. Therefore, an individual must have sufficient sleep to have a better health status and to improve his/her work ability. In addition, the findings here showed that the effect of sleep quality on work ability was mediated by psychological distress. Sahraian and Javadpour [68] also observed significant relationships between sleep quality and general health status as well as psychological distress. In the long term, lack of sleep can lead to psychological distress and low productivity [69]. Consistent findings have shown that sleep duration is positively correlated with psychological well-being [70,71]. Such relations may be explained by the fact that quality sleep can activate the parasympathetic nervous system to secrete growth hormone, which subsequently helps the body to recover from stresses [72]. Supported by several previous studies showing that individuals with psychometric disorders will exhibit poor work ability [19,32], the results in this study found that the positive relationship between sleep quality and work ability was fully mediated by psychological distress. Therefore, participants with sufficient sleep will generally have a lower level of psychological distress and hence better work ability.

For smoking habit, the findings of this study indicated that the effect of smoking on work ability was fully mediated by general health status. Simply put, participants with longer years of smoking will have worse general health status and therefore a decline in work ability. The association between smoking and reported general health was also found by Laaksonen et al. [73]; current smokers among male participants in their study reported poorer general health than non-smokers.

The effect of the frequency of physical activity on work ability was fully mediated by general health status and psychological distress. Epidemiologic and experimental studies have revealed that physical activity can improve cardiorespiratory fitness [74] and reduce the associated risk of coronary heart disease and other physical and psychological related health problems [75,76,77]. Therefore, the positive impact of frequent physical activity on better work ability seems to be the consequence of improved general health and psychological well-being along with routine physical activity.

### 4.5. Individual Competence

Individual/career competence is an important yet rarely studied factor for work ability measurement. The results of this study showed that participants with a higher level of career competence had higher WAI scores. The measurement of individual competence reflected the participants’ professional competences in exercising their duties at work. Thus, participants who were more capable of mastering their work and had clearer career plans and goals had better work ability.

### 4.6. Work-Related Factors

For work-related factors, physical demands directly influenced work ability, while the effect of psychological demands on work ability was partially mediated by all of the health-related factors, viz., general health status, MSD symptoms, and psychological distress. Physically demanding jobs have been consistently reported to be positively associated with a lower WAI [12,19,25,78]. Heavy physical demand is usually due to increased muscular work, long duration of physical work, and poor work postures. In addition, physical work capacity inevitably declines with age, leading to decreased cardiorespiratory capacity and musculoskeletal capacity, which make awkward postures more harmful for the old than for the young [79,80]. The effects of physical demands on work ability were expected to have been mediated by health-related factors; however, this mediation effect was not found in this study. A possible reason may be that the study population in this study was relatively young, and the negative impacts of declining physical capacity with age were not as significant as expected.

The effect of psychological demands on work ability was partially mediated by all the health-related factors. The negative relationship between psychological demands and self-reported general health status was consistent with the findings of Shen et al. [81] that lower general health scores are associated with high psychological demand. For the relationship between psychological demands and MSD symptoms, the study of Aasa et al. [82] reported significant associations between psychological demands and complaints about the neck–shoulder and low-back regions among female ambulance personnel. Such a relationship was hypothesized to be the result of arousal of muscle activity by stress (e.g., psychological demands), subsequently initiating the same processes as in low-load static work. The significant relationship between psychological demands and musculoskeletal symptoms has been consistently reported in the literature [83,84,85,86]. Therefore, construction workers experiencing high psychological demands, such as having to work very fast and perform an excessive amount of work, are more prone to suffering from musculoskeletal symptoms, resulting in lower work ability. The results of the current study are consistent with those of several previous studies that have found that high psychological demand results in psychological distress or depressive symptoms [87,88]. Apart from the mediation effects of psychological demands on work ability through health-related factors, psychological demands also directly affected WAI scores. A systematic review by van den Berg et al. [34] found that a positive association between high mental work demands and poor work ability has been reported in several studies [43,78,89]. Work ability results from interaction between workers and their work; hence, increase in physical and/or psychological work demands will very likely lead to reduced work ability.

The effects of job control and social support on work ability were found to be mediated by general health status. This finding is similar to results reported by Smith et al. [90] on the relationships between job control and health status, where a higher level of job control led to better health status. Bosma et al. [91] reported that a low level of job control at work leads to the development of coronary heart disease. They suggested that providing employees with more variety in work tasks as well as more say at work will be beneficial to employee health. On the relationship between social support and health, Uchino [92] explained that social support affects health outcomes in two possible ways. The first is that social support can facilitate healthier behaviors and greater adherence to medical regiments [93]. The second way is that low levels of social support are linked to psychological processes; including appraisals, emotions and feelings of control [94]. As a result, low levels of social support will lead to higher mortality rates, particularly from cardiovascular disease, as confirmed by epidemiological studies [95].

## 5. Limitations

Despite the promising contributions made by this study, there are some limitations of this study. It is known that with a large sample size of 420, correlations as low as <0.2 are still statistically significant. However, such low values, though statistically proven to be significant, may be regarded as practically insignificant to certain extent by practitioners. Also, this study is somewhat ambitious to include a large number of factors in the model, which might have obscured some points of the study. In addition, the relationship between sleep quality and work conditions was not considered here.

## 6. Practical Implications

In order to formulate pragmatic strategies for sustaining the construction workforce in Hong Kong, the findings of this study can be used as the basis for formulating an effective intervention program for the construction industry, with the aim of promoting healthy behavior to construction workers, lowering their work demands, and increasing their influence and support in the workplace. The approach of intervention mapping (IM) has been increasingly used as an effective method for complex health promotion interventions, given its systematic approach to program planning along with the inclusion of robust theories, empirical evidence, and practical information [96,97]. The identified significant factors in this study can therefore be applied as part of the empirical information for the intervention program formulation so as to improve construction workers’ low work ability due to the negative impacts of the individual factors and work-related factors on their health. The effectiveness of the program can be evaluated in a randomized controlled trial to measure and compare the outcomes after receiving the interventions. The outcomes for evaluation should be the work ability of the workers as well as the different individual and work-related factors examined in this study. To facilitate the implementation of the program, a feedback loop should be established at different steps of the intervention program, such that any problem that appears during the intervention can be effectively identified and hence immediately rectified to continuously improve the intervention program. In summary, the findings of this study would guide the industry in the formulation of pragmatic and realistic strategies to cope with the acute shortage of the construction workforce in Hong Kong by considering the individual and work-related factors significantly affecting the work ability of the workers.

## 7. Conclusions

The findings of this study suggested that the work ability of the construction workers was significantly affected by different lifestyle and work-related factors, including the physical and psychological demands of their work, job control, and social support. Except for physical demands and individual competence, all the effects of individual and work-related factors on work ability were fully or partially mediated by health-related factors, indicating that the health of the workers was a determining factor for their work ability and for possible retention of workers in the industry. Work ability was found to decline along with deteriorating health of participants due to poor sleep quality, having a smoking habit, lack of physical exercise, high psychological demands of the work, as well as low levels of job control and social support from work. In addition, poor work ability was directly associated with high physical demands of work and low competence of participants. The results obtained in this study provide solid evidence of the impact of various individual and work-related factors on the work ability of Hong Kong construction workers. The influence of these significant factors should be taken into consideration when formulating comprehensive intervention programs to better retain the valuable construction workforce in Hong Kong.

## Figures and Tables

**Table 1:** The Work Ability Index (WAI) scores of workers of different occupations in various countries (from 2000 to 2016).

Authors	Objectives	Study Population	Mean WAI	Country	Findings
Kloimüller et al. [41]	To analyze the relationship among age, WAI, and subjective stressors in bus drivers	369 bus drivers, mean age of 43.9 years	36.8	Finland	A low correlation between WAI and age was determined, and subjective stressors and stress symptoms were strongly related to WAI.
Pohjonen [18]	To analyze the relationship among WAI, individual characteristics, and work-related factors in home care workers	636 female blue-collar home care workers, mean age of 42.3 years	37.7	Finland	WAI was strongly associated with age and musculoskeletal and psychosomatic symptoms. Meanwhile, ergonomics, possibilities to control one’s own work, time pressure, and time management were the work factors that predict work ability.
Sjögren-Rönkä et al. [42]	To investigate the relationship between work ability and the prerequisites of physical and psychological functioning	88 office workers, mean age of 45.7 years	Median ≤45 years—43 ≥46 years—40	Finland	Work ability was generally affected by the physical prerequisites of functioning; high-intensity musculoskeletal symptoms had the most substantial negative effect on work ability.
Lin et al. [40]	To assess the work ability of workers in western China	3939 manual workers, 3963 professional and clerical workers, and 2313 semi-skilled workers, mean age of 36.8 years	Manual workers—38.1, Professional and clerical workers—38.6, Semi-skilled workers—39.3	China	The WAI scores decreased with age and varied among different occupations. The mean WAI declined drastically after the age of 35 for manual workers and 45 years for professional and clerical workers.
Alavinia et al. [5]	To evaluate the influence of work-related factors and individual characteristics on work ability among Dutch construction workers	19,507 Dutch construction workers, mean age of 44.1 years	40.9	Netherlands	Physical workload and psychosocial factors at work explained 22% of the variability in work ability. Awkward back posture, static work postures, repetitive movements, and lack of support at work imposed the most substantial influence on work ability.
van den Berg et al. [16]	To investigate the associations of psychosocial factors at work and lifestyle with health and work ability	1141 white-collar workers in commercial services, median age of 35.7 years	41.1	Netherlands	Work ability was strongly associated with psychosocial factors at work among white-collar workers. Moreover, the influence of an unhealthy lifestyle had a more substantial effect on the work ability of older workers than that of younger workers.
Alavinia et al. [28]	To analyze the effects of work-related factors and individual characteristics on work ability and the predictive value of work ability on the receipt of a work-related disability pension in a longitudinal study (23 months)	850 construction workers, mean age of 48.4 years	38.7	Dutch	All work-related risk factors were associated with low work ability. Participants with moderate or poor work ability were highly predictive for receiving disability pension.
van den Berg et al. [43]	To evaluate the association between decreased work ability and productivity loss at work, as well as the influence of high physical and psychosocial workload	10,542 workers in 49 different companies in the Netherlands, mean age of 44 years	7–27 (3.4%) 28–36 (16.4%) 37–43 (47.4%) 44–49 (32.8%)	Netherlands	A significant interaction between decreased work ability and lack of job control with productivity loss at work was reported.
Mazloumi et al. [44]	To analyze the relationship between WAI and its association with psychosocial factors among workers in the petrochemical industries in Iran	420 officers (97), laboratory technicians (36), fire fighters (27), gas-field workers (160) and maintenance workers (100) in an Iranian petrochemical industry, mean age of 40.2 years	39.1	Iran	WAI was significantly associated with psychosocial factors. Skill discretion, coworker support, and supervisor support were positively associated with the mean WAI score. By contrast, job demands, job strain, and job insecurity exhibited inverse associations.
El Fassi et al. [45]	To compare the assessment of work ability based on the seven-item WAI questionnaire with that based on the first item of the WAI questionnaire	12,839 workers in Luxembourg, mean age of 47 years	41.0	Luxembourg	The risk of having moderate or poor work ability was increased with age, being overweight, decline in health status, having a physically demanding job, and working in a large company. Moreover, using the single item for work ability measurement generated results similar to that using seven items.
Han et al. [46]	To evaluate the association of work-related factors and migration characteristics with work ability among Chinese migrant workers	907 Chinese migrant workers in the Pearl River Delta, mean age of 30.3 years	40.1	China	Social support was the migration characteristic that was significantly associated with WAI. Accordingly, WAI can be increased by improving the physical and psychosocial work factors of migrant workers.
Roelen et al. [47]	To investigate the association of WAI with premature work exit by means of disability pension, unemployment, or early retirement	11,537 male construction workers, mean age of 45.5 years	40.1	Netherlands	The WAI scores were associated with the risk of premature work exit with disability pension but not of unemployment and early retirement. Moreover, WAI effectively identified the risk of construction workers with <50 years of age receiving disability pension.
Rutanen et al. [48]	To investigate the effectiveness of a six-month physical exercise program among women with menopausal symptoms	45 (intervention group) and 44 (control group) women with menopausal symptoms, mean ages of 54.8 (intervention group) and 54.1 (control group)	38.3 (intervention group) 38.7 (control group)	Finland	The increase in WAI was significantly higher among the intervention group than the control group. Physical exercise intervention had positive short-term and long-term effects on work ability.
Lian et al. [29]	To analyze the effects of insomnia and sleep duration on poor work ability	2820 Chinese manufacturing workers, with the age ranges of <30 (48.4%), 30–40 (18.7%), 40–50 (24.2%), and >50 (8.8%)	≤36 (20.6%) ≥37 (79.4%)	China	Insomnia and short sleep duration were independently associated with poor work ability. Participants with insomnia with < 5 h sleep duration were at the highest risk of poor work ability.
Mache et al. [49]	To analyze the associations of job performance and organizational and individual resources with work ability of doctors working in psychiatric hospitals in Germany	248 physicians, most of their ages in 26–35 years (54%)	39.2	Germany	Significant associations between the participants’ work engagement, organizational factors, and work ability were observed. The individual factors of gender, age and marital status were also significantly related to WAI.
Wilke et al. [50]	To assess the work ability and work-related physical activity of employees in a chemical company in Germany	148 employees, mean age of 40.9 years	White-collar workers—43 Blue-collar workers—40	Germany	Occupation (i.e., white-collar versus blue-collar workers) and work-related physical activity, but not age, showed significant differences in work ability of the participants.
Gharibi et al. [51]	To investigate the association of work-related stress with work ability among Iranian workers	449 Iranian workers from five different working sectors, mean age of 34.1 years	38.0	Iran	One-third of the participants had work ability below 37. Moreover, a significant correlation between work-related stress and WAI was observed.

**Table 2:** Average scores for WAI and its subscales.

WAI and its Subscales	Mean	SD	Range
Current work ability compared with the lifetime best	8.89	1.46	3–10
Work ability in relation to the physical and mental demands of the job	9.02	1.28	3–10
Number of current diseases diagnosed by a physician	6.10	1.24	1–7
Estimated work impairment due to diseases	5.86	0.41	2–6
Sick leave during the past year (12 months)	4.65	0.55	1–5
Own prognosis of work ability 2 years from now	6.73	0.86	4–7
Mental resources	3.87	0.39	1–4
**Overall WAI**	45.12	3.38	27–49

**Table 3:** Descriptive data for lifestyle factors, health factors, individual competencies, and work-related factors for the 420 participants.

Variables	Mean	SD	Min	Max
**Lifestyle Factors**				
Alcohol consumption per week (mL)	377.99	1286.10	0	15,360
Years of alcohol drinking	2.78	7.91	0	50
Cigarette consumption per day	3.77	6.88	0	30
Years of smoking	4.15	9.61	0	51
Frequency of physical activity	3.29	1.39	1	5
Strength of physical activity	3.15	1.29	1	5
**Health-related Factors**				
General health status	4.39	0.86	1	5
MSD symptoms in last 12 months	2.04	2.82	0	27
Mild	1.08	1.39	0	9
Moderate	0.52	0.95	0	9
Severe	0.44	0.94	0	9
Psychological distress	28.46	2.73	13	30
**Individual competence**	4.29	0.69	1.52	5
Reflection on motivation	4.56	0.56	2	5
Reflection on qualities	4.42	0.75	1	5
Networking	4.04	0.78	1	5
Self-profiling	4.30	0.94	1	5
Work exploration	4.28	1.03	1	5
Career control	4.18	1.13	1	5
**Work-related Factors**				
Physical demands	12.93	2.66	5	20
Psychological demands	23.04	6.97	12	46
Job control	74.68	13.70	26	94
Supervisor/company support	14.63	2.14	5	16
Co-worker support	13.83	3.00	4	16

**Table 4:** Pearson’s correlation analysis for different individual and work-related factors.

	WAI	GHS	MSD	PD	AC	YoAD	SA	YoS	FoPE	SoPE	SQ	IC	PhyD	PsyD	JC	S/CS	CoS
**WAI**	–	0.59 ***	−0.43 **	0.38 ***	−0.14 **	−0.12 *	−0.23 ***	−0.25 ***	0.26 ***	0.23 ***	0.16 **	0.4 ***	−0.13 **	−0.52 ***	0.34 ***	0.39 ***	0.33 ***
**GHS**		–	−0.29 ***	0.45 ***	−0.14 **	−0.2 ***	−0.26 ***	−0.33 ***	0.47 ***	0.5 ***	0.28 ***	0.46 ***	−0.17 **	−0.49 ***	0.46 ***	0.55 ***	0.4 ***
**MSD**			–	−0.19 ***	0.03	−0.003	0.14 **	0.13 **	−0.06	−0.05	−0.12 *	−0.06	0.12 *	0.18 ***	0.04	−0.13 *	−0.14 **
**PD**				–	−0.19 ***	−0.04	−0.13 *	−0.08	0.26 ***	0.16 **	0.24 ***	0.36 ***	−0.09	−0.43 ***	0.27 ***	0.32 ***	0.25 ***
**AC**					–	0.57 ***	0.33 ***	0.28 ***	−0.16 **	−0.12 *	−0.11 *	−0.15 **	−0.02	0.16 **	−0.05	−0.09	−0.09
**YoAD**						–	0.26 ***	0.34***	−0.21 ***	−0.17 ***	−0.08	−0.16 **	0.02	0.2 ***	−0.13 **	−0.21 ***	−0.22 ***
**SA**							–	0.76 ***	−0.34 ***	−0.25 ***	−0.09	−0.21 ***	0.18 ***	0.29 ***	−0.14 **	−0.23 ***	−0.15 **
**YoS**								–	−0.39 ***	−0.35 ***	−0.05	−0.23 ***	0.17 ***	0.34 ***	−0.22 ***	−0.31 ***	−0.17 ***
**FoPE**									–	0.73 ***	0.11 *	0.45 ***	−0.11 *	−0.36 ***	0.4 ***	0.36 ***	0.18 ***
**SoPE**										–	0.08	0.47 ***	−0.14 **	−0.27 ***	0.44 ***	0.37 ***	0.17 **
**SQ**											–	0.07	−0.02	−0.21 ***	0.02	0.11 *	0.13 **
**IC**												–	−0.05	−0.4 ***	0.72 ***	0.56 ***	0.31 ***
**PhyD**													–	0.42 ***	−0.07	−0.11 *	−0.05
**PsyD**														–	−0.41 ***	−0.47 ***	−0.37 ***
**JC**															–	0.58 ***	0.29 ***
**S/CS**																–	0.43 ***
**CoS**																	–

**GHS**: General Health Status, **MSD**: No. of MSD Symptoms, **AC**: Alcohol Consumption, **YoAD**: Years of Alcohol Drinking, **SA**: Smoking Amount, **YoS**: Years of Smoking, **FoPE**: Frequency of Physical Exercise, **SoPE**: Strength of Physical Exercise, **PD**: Psychological Distress, **SQ**: Sleep Quality, **IC**: Individual Competence, **PhyD**: Physical Demands, **PsyD**: Psychological Demands, **JC**: Job Control, **S/CS**: Supervisor/Company Support, **CoS**: Co-worker Support. Note. * *p* < 0.05, ** *p* < 0.01, *** *p* < 0.001.

**Table 5:** Results of hypothesis testing.

Hypothesis		Results
**H1**	Lifestyle factors will be associated with the WAI.	
**H1.1**	Alcohol consumption will be negatively associated with the WAI.	Not supported
**H1.2**	Smoking habit will be negatively associated with the WAI.	Supported
**H1.3**	Leisure-time physical activity will be positively associated with the WAI.	Supported
**H1.4**	Sleep quality will be positively associated with the WAI.	Supported
**H2**	The associations between the lifestyle factors and the WAI will be mediated by health-related factors.	Partially supported
**H2.1**	Self-reported general health status will be positively associated with the WAI.	Supported
**H2.2**	Self-reported MSD symptoms will be negatively associated with the WAI.	Supported
**H2.3**	Self-reported psychological distress will be negatively associated with the WAI.	Supported
**H3**	Individual competence will be positively associated with the WAI.	Supported
**H4**	Work demand factors will be associated with the WAI.	
**H4.1**	Physical demands will be negatively associated with the WAI.	Supported
**H4.2**	Psychological demands will be negatively associated with the WAI.	Supported
**H4.3**	*Job control is positively associated with the WAI.*	Supported
**H4.4**	*Social support is positively associated with the WAI.*	Supported
**H5**	The associations between the work-related factors and the WAI will be mediated by individual health-related factors.	Partially supported
